# Multi-site cholera surveillance within the African Cholera Surveillance Network shows endemicity in Mozambique, 2011–2015

**DOI:** 10.1371/journal.pntd.0005941

**Published:** 2017-10-09

**Authors:** Cynthia Semá Baltazar, José Paulo Langa, Liliana Dengo Baloi, Richard Wood, Issaka Ouedraogo, Berthe-Marie Njanpop-Lafourcade, Dorteia Inguane, Jucunu Elias Chitio, Themba Mhlanga, Lorna Gujral, Bradford D. Gessner, Aline Munier, Martin A. Mengel

**Affiliations:** 1 Instituto Nacional de Saúde, Surveillance Department, Maputo, Mozambique; 2 Instituto Nacional de Saúde, Microbiology Laboratory, Maputo, Mozambique; 3 Agence de Médecine Préventive, Enteric Diseases Program, Paris, France; 4 National Public Health Directorate, Epidemiology Department, Ministry of Health, Maputo, Mozambique; Massachusetts General Hospital, UNITED STATES

## Abstract

**Background:**

Mozambique suffers recurrent annual cholera outbreaks especially during the rainy season between October to March. The African Cholera Surveillance Network (Africhol) was implemented in Mozambique in 2011 to generate accurate detailed surveillance data to support appropriate interventions for cholera control and prevention in the country.

**Methodology/Principal findings:**

Africhol was implemented in enhanced surveillance zones located in the provinces of Sofala (Beira), Zambézia (District Mocuba), and Cabo Delgado (Pemba City). Data were also analyzed from the three outbreak areas that experienced the greatest number of cases during the time period under observation (in the districts of Cuamba, Montepuez, and Nampula). Rectal swabs were collected from suspected cases for identification of *Vibrio cholerae*, as well as clinical, behavioral, and socio-demographic variables. We analyzed factors associated with confirmed, hospitalized, and fatal cholera using multivariate logistic regression models.

A total of 1,863 suspected cases and 23 deaths (case fatality ratio (CFR), 1.2%) were reported from October 2011 to December 2015. Among these suspected cases, 52.2% were tested of which 23.5% were positive for *Vibrio cholerae* O1 Ogawa. Risk factors independently associated with the occurrence of confirmed cholera were living in Nampula city district, the year 2014, human immunodeficiency virus infection, and the primary water source for drinking.

**Conclusions/Significance:**

Cholera was endemic in Mozambique during the study period with a high CFR and identifiable risk factors. The study reinforces the importance of continued cholera surveillance, including a strong laboratory component. The results enhanced our understanding of the need to target priority areas and at-risk populations for interventions including oral cholera vaccine (OCV) use, and assess the impact of prevention and control strategies. Our data were instrumental in informing integrated prevention and control efforts during major cholera outbreaks in recent years.

## Author summary

Cholera is a major public health problem in many countries in sub-Saharan Africa. In Mozambique, annual outbreaks occur but the place and time may vary. Africhol was implemented in Mozambique in 2011 to generate more detailed information on disease burden and characteristics to support appropriate interventions for cholera control and prevention in the country. The study was conducted in six different zones, where patients with cholera symptoms seeking care at a health facility were asked questions on socio-demographic characteristics, their symptoms, and behaviors that may increase cholera risk. Stool samples were also taken to test for the presence of cholera infection (*Vibrio cholerae*). Among the 1,863 patients, more than half were tested for cholera, and among those tested, less than one in four was infected with the pathogen. About 1% of patients died from cholera. Our study helps to understand the burden of cholera in different areas of the country, and the characteristics of the people infected. It is important to continue the surveillance of this disease to choose the most appropriate control and preventive interventions, and to apply them in precisely the right place.

## Introduction

Cholera is an acute, diarrheal illness caused by infection of the intestine with the bacterium *Vibrio cholerae*. Its epidemics have been continuously reported from Southern Africa since its reintroduction on the continent in 1970 [1, 2]. Today, cholera remains a significant cause of morbidity and mortality, and a key indicator of lack of adequate infrastructure and structural development, specifically an insufficient supply of drinking water and inadequate sanitation [3, 4]. While there has been significant work done in Africa to quantify the magnitude of cholera as a public health problem in recent years, individual and population characteristics in specific settings remain ill defined.

Since 1973, Mozambique has reported cholera almost yearly. Cholera in Mozambique is an endemic disease with seasonal epidemic peaks. The usual cholera season is between January and March with annual incidences ranging from 0 to 211 per 100,000 population with periodically high case-fatality ratios (CFRs) [5–9].

Disease burden data are critical for making evidence-based decisions on public health interventions, including water, sanitation, and hygiene (WASH) and use of oral cholera vaccine (OCV). In Mozambique, epidemiological surveillance data are used to identify districts at risk of cholera and to identify contributing factors such as an insufficient supply of drinking water, sanitation practices harmful to health (such as open defecation, using dirty or contaminated water, and handling and selling unhygienic food), and natural disaster [5].

Africhol is a multi-centric project that was launched in 2009 with funding from the Bill & Melinda Gates Foundation. It consists of a consortium of 11 African countries and non-governmental organizations, seeking to collect epidemiological and microbiological information regarding the occurrence of cholera in Africa to better inform intervention strategies (http://amp-vaccinology.org/activity/cholera-in-africa)[10].

In Mozambique, the Africhol project was implemented in 2011, to conduct prospective surveillance in dedicated surveillance zones with the main objective of assessing cholera burden. The current study presents the results from five years of surveillance in terms of disease burden, population characteristics, and risks factors in specific surveillance zones to support appropriate interventions for cholera control and prevention.

## Materials and methods

### Ethics statement

The surveillance protocol was approved by the Mozambican National Bioethics Committee for Health. The informed oral consent was obtained from all participants and documented by a specific question on the case report form. The Ministry of Health (MOH) considered that the Africhol project was integrated in their national epidemic disease surveillance and response and subject to the laws and regulations, and thus did not require written consent. For minors below 18 years, informed oral consent was obtained from parents, guardians, or next of kin, on behalf of the child. Oral consent information provided enough details to the study participants about the stool and blood sample collection process as well as the planned use of these specimens.

### Study population

In 2011, the project was first implemented in two enhanced surveillance zones: Beira city in Sofala province, and Mocuba district in Zambézia province. In 2013, a third surveillance zone was established in Pemba city, Cabo Delgado province, following several cholera outbreaks there. These three areas were among the 8 districts out of the country's 145 districts with the highest number of cholera cases (>1000) reported to the Ministry of Health from 2009 to 2011 [5].

Beira city is the third largest city in Mozambique with a population of 463.442. Mocuba district includes 404.749 inhabitants and is rural area. Pemba City, with a population of 218.152, is the capital city of northernmost Cabo Delgado province. It has preserved largely rural characteristics with low population density.

The enhanced surveillance zones were selected based on: a high incidence of cholera during the previous five years; a sufficient large population denominator to determine incidence; a history of reliably providing surveillance reports on suspected cholera cases to the National MOH—Surveillance System; reliable health care access for the local population and diagnostic facilities to allow case identification. In these areas we included all health centers that treated the local population for severe diarrhea and collected additional data through community investigations. Usually, specific cholera treatment centers (CTC) were open only during cholera outbreak periods when declared by health provincial authorities. According to the area, the delay to open CTCs varied. Outside outbreaks patients with severe diarrhea were treated at general health centers or hospitals.

Africhol was integrated into the routine cholera surveillance and provided additional support beyond that supported by the MOH. For example, Africhol provided funding for specific additional staff such as a country focal point, a coordinator and a surveillance officer for each zone who ensured the proper conduct of the study, data collection, data quality and supervision of data entry. Also, in the enhanced surveillance zones, whenever possible most suspected cases were culture-tested, while in the routine system, only a few suspected cases at the start, during and at the end of an epidemic were usually tested for cholera. Africhol provided repeated refresher training for laboratory staff. Finally, Africhol provided standard case-report forms (CRFs) for data collection, laboratory standard operating procedures, support for specimen transport, support for serotyping, and technical assistance from the Africhol international team.

**Fig 1:**
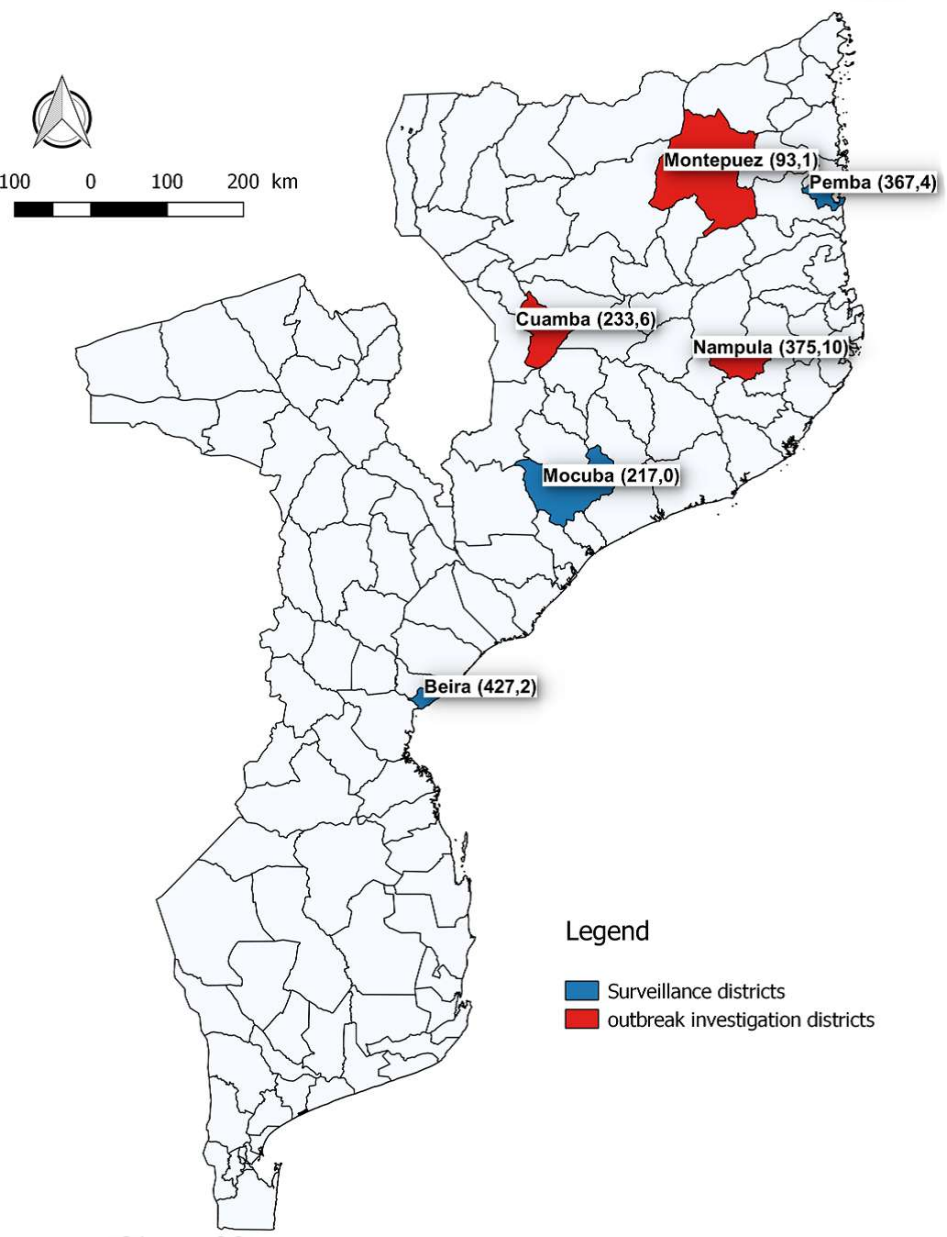
Africhol surveillance zones and outbreak sites in Mozambique. Note: Numbers in brackets are (cases, deaths).

Data was collected prospectively through Africhol since its implementation in October 2011 until December 2015 in the three surveillance zones of Beira, Mocuba, and Pemba (Fig 1). This information was collected year round [11]. In addition, outside these enhanced surveillance zones, and using the same Africhol tools, we also investigated individual cholera outbreaks in three sites that experienced the greatest number of cases during the same time period, in Montepuez district (Cabo Delgado province) during February 2012, Cuamba district (Niassa province) during January-February 2012, and Nampula city (Nampula Province) every year from 2012 to 2015, as well as a few cases from other zones (mainly from Tete, Quelimane and Lichinga districts during outbreaks occurring in 2015). Those cases were investigated mainly from CTCs.

### Case definition and data collection

For Africhol, we defined a suspected case as an individual at least one year of age or older, with acute watery diarrhea and dehydration or must have died from acute watery diarrhea. A cholera case was considered confirmed when *Vibrio cholerae O1* was isolated from a stool sample or rectal swab specimen by microbiological culture.

These definitions differed slightly from those used by the national MOH routine surveillance system for which a suspected case is defined as any patient aged two years or older, with acute watery diarrhea and abdominal pain, and profuse watery stools (type rice water stools), with vomiting or rapid dehydration. According to MOH definitions, once a case of cholera in a neighborhood has been confirmed, suspected cases are then considered cholera for a varying period of time, ranging from two to six months depending on the district.

Africhol data were collected through a CRF which included demographic, clinical, behavioral, and laboratory information. The rainy season was defined as the months of October to March, and the dry season from April to September.

### Laboratory methods

Rectal swabs from suspected cholera patients were collected by health staff for laboratory investigation, and then transported from the health unit to the regional or provincial laboratories in Cary Blair transport media at room temperature conditions by a private courier services company once a week. In local laboratories samples were pre-enriched in alkaline peptone water and pre-incubated at 35–37°C for six to eight hours, followed by *V*. *cholerae* diagnostic tests carried out by sub-culturing on thiosulphate citrate bile salts sucrose agar (TCBS). After pre-processing at provincial level, the isolates cultured positive (showing yellow colonies) on TCBS were sent to the National Microbiology Laboratory at the Instituto Nacional de Saúde (INS) in the capital Maputo for further confirmation and quality control by standard biochemical tests and serology using polyvalent, anti-Ogawa, and anti-Inaba antisera. An aliquot of *V*. *cholerae* isolates was stored in the INS lab at -80°C and a copy was sent to the National Institute for Communicable Diseases (NICD) in South Africa for quality control and molecular subtyping and results were previously published elsewhere [12].

Blood samples were systematically taken for malaria and HIV testing in all sites from those patients that were in conditions to consent and accepted to have an HIV test. HIV counseling and testing were performed according to Mozambique’s national guidelines, which include confidentiality, counseling, and informed consent. Current guidelines for rapid testing call for a two test serial testing algorithm that screens with Determine HIV-1/2 (Alere, USA), and confirmation with Uni-Gold HIV (Trinity Biotech, Ireland). Malaria tests were performed by a health professional according to the national guidelines using one of several rapid tests available to the National Health Service.

### Data analysis

#### Outcomes of interest

- The cholera burden in each zone was measured through the number of suspected, tested, and confirmed cases. We defined confirmed cholera as either 1) culture positive result and oxidase positive result; or 2) a culture positive result and identified serotype (i.e. Ogawa, which was the only serotype detected in our study).
- The annual incidence of suspected cases was defined as the number of cases divided by 10,000 population. For outbreak sites, the attack rate per outbreak was defined as the number of cases per 10,000. The source of population data was the Mozambican National Institute of Statistics (INE, http://www.ine.gov.mz/estatisticas/estatisticas-demograficas-e-indicadores-sociais/projeccoes-da-populacao), which provided population projections for each district since the 2007 population census.
- The number of patients hospitalized (defined as admission of at least one night for treatment of cholera) was measured across sites, and factors associated with hospitalization were studied.
- The number of deaths from cholera at the treatment facility was measured across sites, and factors associated with death were studied (see method below).
- The cholera case-fatality ratio (CFR suspected) was calculated as the number of deaths attributable to cholera divided by the number of suspected cases. The CRF for confirmed cases was also calculated (CFR confirmed), with the number of etiologically confirmed cases as denominator.

#### Patients' characteristics and difference between sites

Patients’ socio-demographic, clinical, and behavioral characteristics were compared between sites. Proportions were compared using the Chi2 test or Fisher’s exact test and continuous variables were compared using analysis of variance (ANOVA) or the Kruskall-Wallis test, as appropriate. Differences between groups were considered significant if P<0.05.

#### Factors associated with confirmed cholera

Using a test-negative case-control design, we analyzed the factors associated with confirmed cholera. From all suspected cholera patients who were tested for cholera at the laboratory, cases were defined as those ‘confirmed cholera’ based on the definition above and controls were those culture-negative. We used logistic regression models for univariate and multivariate analysis of factors associated with confirmed cholera. The different steps included: i) univariate analysis; ii) univariate analysis using surveillance zone as confounding factor; iii) all relevant, non-collinear variables with P values<0.20 in step ii were entered into a multivariate model that was adjusted for gender and age. Cases from two zones–Cuamba and Montepuez–were not entered into the multivariate model because of lack of testing (only one case tested in each of these zones). Similarly, we did not include the 22 cases identified in 2011 in the multivariate model because surveillance started only at the end of that year which would have introduced a selection bias in our analysis. Multivariate logistic regression was done by removing variables one by one in a manual backward procedure using likelihood ratio tests at each step. Variables were kept in the final model if P<0.05. Age and gender were forced into the model as adjusting variables. Interactions were tested in the final model. A similar procedure was used to assess factors associated with hospitalization and death.

## Results

### Socio-demographic characteristics of cholera suspected cases

**Fig 2:**
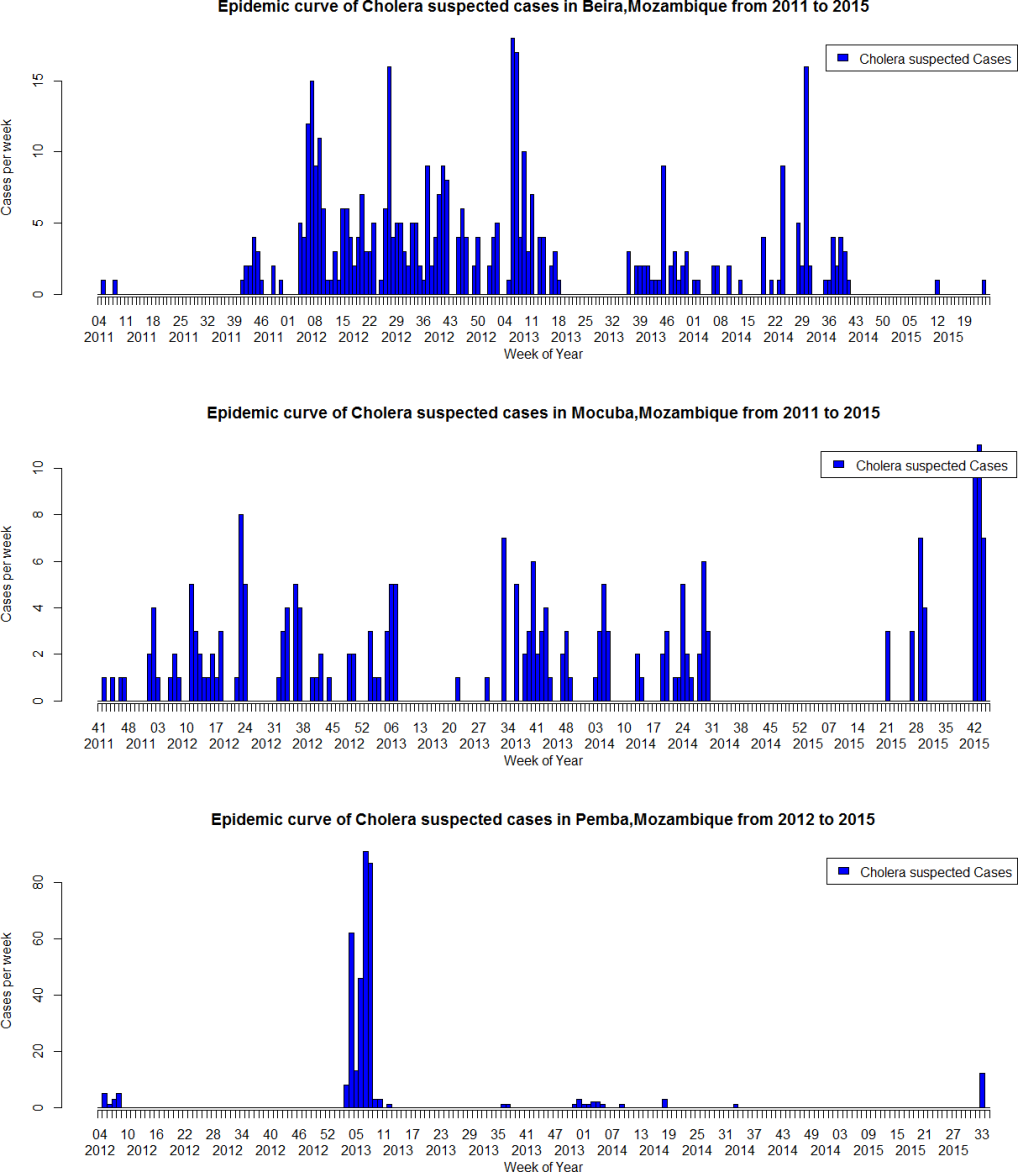
Epidemic curves, Africhol surveillance zones, Mozambique, 2011–2015: Beira City, Mocuba District, and Pemba City.

**Fig 3:**
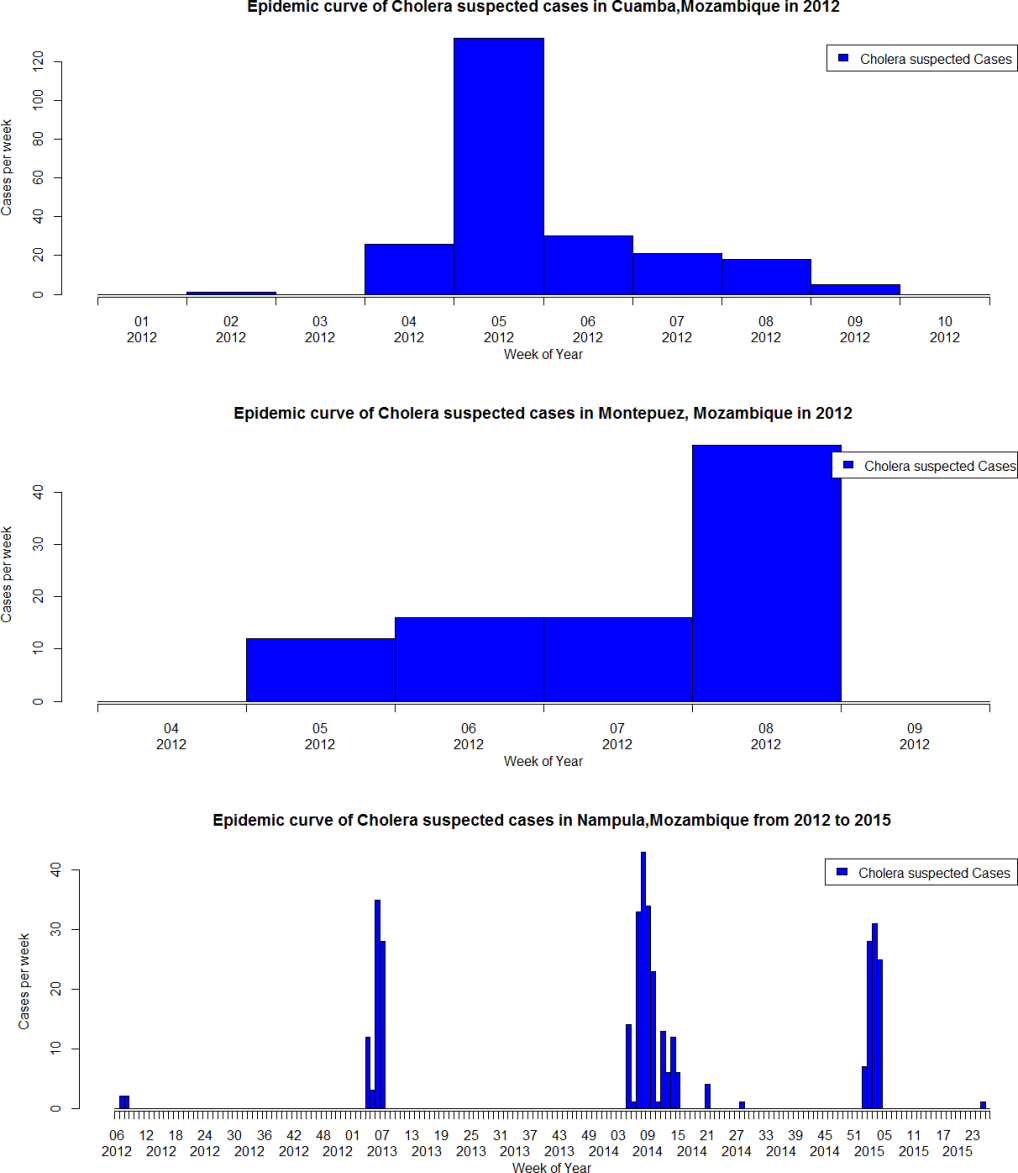
Epidemic curves, Africhol outbreak sites, Mozambique, 2011–2015: Cuamba District, Montepuez District and Nampula City.

From October 2011 to December 2015, a total of 1,863 suspected cholera cases were reported through the Africhol surveillance system. Among them, 1,010 (54.2%) were reported from the surveillance zones (Fig 2) and 853 (45.8%) from cholera outbreaks investigated in Cuamba, Montepuez, and Nampula (Fig 3). Additionally, a few cases were reported episodically from other zones, including mainly from Tete, Quelimane, and Lichinga during outbreaks occurring in 2015 (n = 151). Overall, the majority of cases (81.7%) were reported during the rainy season (October-March), especially in outbreak zones compared to surveillance sites (96.0% vs. 69.7%, p<0.001). Male-to-female sex ratio was 1.14 and varied between sites, from 1.49 in Nampula to 0.75 in Montepuez. The median age was 20 years (interquartile range (IQR), 9–32). There was a similar age distribution in all zones, except in Beira, which had the lowest median age (nine years) with the highest proportion of suspected cases under or equal to five years (38.4%, p<0.001) (Table 1). There were 101 children aged 12–23 months–mainly in Beira, thus representing 33% of the overall ‘below 5 years-old” age category (308).

**Table 1:** Socio-demographic characteristics of cholera suspected cases, 2011–2015, Mozambique.

		Surveillance Zones			Outbreak Sites			
Characteristics	All zones	Beira	Mocuba	Pemba	Cuamba	Montepuez	Nampula	P value
	N = 1863	N = 427	N = 217	N = 367	N = 233	N = 93	N = 375	
**Year**								<0.001
2011	22 (1.2)	18 (4.2)	4 (1.8)	0	0	0	0	
2012	640 (34.3)	223 (52.2)	69 (31.8)	14 (3.8)	233 (100)	93 (100)	4 (1.1)	
2013	579 (31.1)	119 (27.9)	58 (26.7)	321 (87.4)	0	0	81 (21.6)	
2014	316 (17.0)	65 (15.2)	41(18.9)	16 (4.4)	0	0	193 (51.5)	
2015	306 (16.4)	2 (0.5)	45 (20.7)	16 (4.4)	0	0	97 (25.9)	
**Season**								
Dry (Apr.-Sept.)	340 (18.3)	181 (42.4)	100 (46.1)	25 (6.8)	0	0	30 (8.0)	<0.001
Rainy (Oct.-Mar.)	1523 (81.7)	246 (57.6)	117 (53.9)	342 (93.2)	233 (100)	93 (100)	345 (92.0)	
***Sex***								0.01
Male	988 (53.0)	233 (54.6)	112 (51.6)	188 (51.2)	112 (48.1)	40 (43.0)	224 (59.7)	
Female	870 (46.7)	192 (44.9)	105 (48.4)	179 (48.8)	121 (51.9)	53 (57.0)	150 (40.0)	
Missing	5 (0.3)	2 (0.5)	0	0	0	0	1 (0.3)	
**Age**								
**Median age in yrs (IQR)**	20 (9–32)	9 (2–27)	21 (11–35)	22 (15–32)	22 (12–36)	25 (16–40)	22 (15–31)	
**Age group**								<0.001
0–5	308 (16.5)	164 (38.4)	31 (14.3)	12 (3.3)	21 (9.0)	4 (4.3)	28 (7.5)	
6–15	387 (20.8)	79 (18.5)	45 (20.8)	82 (22.3)	54 (23.2)	18 (19.3)	69 (18.4)	
16–25	478 (25.6)	60 (14.1)	48 (22.1)	121 (33.0)	68 (29.2)	28 (30.1)	132 (35.2)	
26–35	286 (15.3)	53 (12.4)	36 (16.6)	67 (18.3)	30 (12.9)	12 (12.9)	75 (20.0)	
36–45	181 (9.7)	36 (8.4)	28 (12.9)	31 (8.4)	24 (10.3)	13 (14.0)	39 (10.4)	
>45	188 (10.1)	30 (7.0)	25 (11.5)	43 (11.7)	36 (15.4)	18 (19.4)	27 (7.2)	
Missing	35 (1.9)	5 (1.2)	4 (1.8)	11 (3.0)	0	0	5 (1.3)	
**Health Facility**								<0.001
Cholera Treatment Center	1179 (63.3)	10 (2.3)	30 (13.8)	340 (92.7)	226 (97.0)	92 (98.9)	363 (96.8)	
Emergency Room	199 (10.7)	152 (35.6)	9 (4.2)	19 (5.2)	0	0	0	
Inpatient department	105 (5.6)	5 (1.2)	91 (41.9)	6 (1.6)	1 (0.4)	0	1 (0.3)	
Outpatient department	178 (9.6)	94 (22.0)	74 (34.1)	0	5 (2.2)	1 (1.1)	1 (0.3)	
Pediatric clinic	159 (8.5)	156 (36.5)	3 (1.4)	0	0	0	0	
Other	37 (2.0)	8 (1.9)	10 (4.6)	0	1 (0.4)	0	8 (2.1)	
Missing	6 (0.3)	2 (0.5)	0	2 (0.5)	0	0	2 (0.5)	

IQR: Interquartile range

The annual district-level incidence of suspected cases in surveillance zones ranged from 0.04 per 10,000 in Beira (2015) to 29.7 in Pemba (2013). In outbreak sites, the maximum attack rate was seen in Nampula city with 16.1 cases per 10,000 during the 2015 outbreak (Table 2).

**Table 2:** Estimated cholera incidence rates by site, 2011–2015, Mozambique.

	Site	Year	Suspected cases	Population	Incidence (/10,000) by year for surveillance zones, attack rate for outbreak sites
Surveillance zones	Beira	2011*	18	454,003	1.6
		2012	223	456,005	4.9
		2013	119	457,799	2.6
		2014	65	459,430	1.4
		2015	2	460,904	0.04
	Mocuba	2011*	4	344,822	0.5
		2012	69	355,299	1.9
		2013	58	365,707	1.6
		2014	41	375,934	1.1
		2015	45	385,902	1.2
	Pemba	2012**	130	174,572	7.5
		2013**	541	182,446	29.7
		2014	16	190,741	0.8
		2015**	191	199,457	9.6
Outbreak sites	Cuamba	2012	233	222,800	10.5
	Montepuez	2012**	277	217,736	12.7
	Nampula	2012	4	571,284	0.07
		2013**	491	588,669	8.3
		2014**	454	605,760	7.5
		2015**	1003	622,423	16.1

*Enhanced surveillance started in October 2011.

**National MOH data was used to complement Africhol data for specific districts and years.

**Fig 4:**
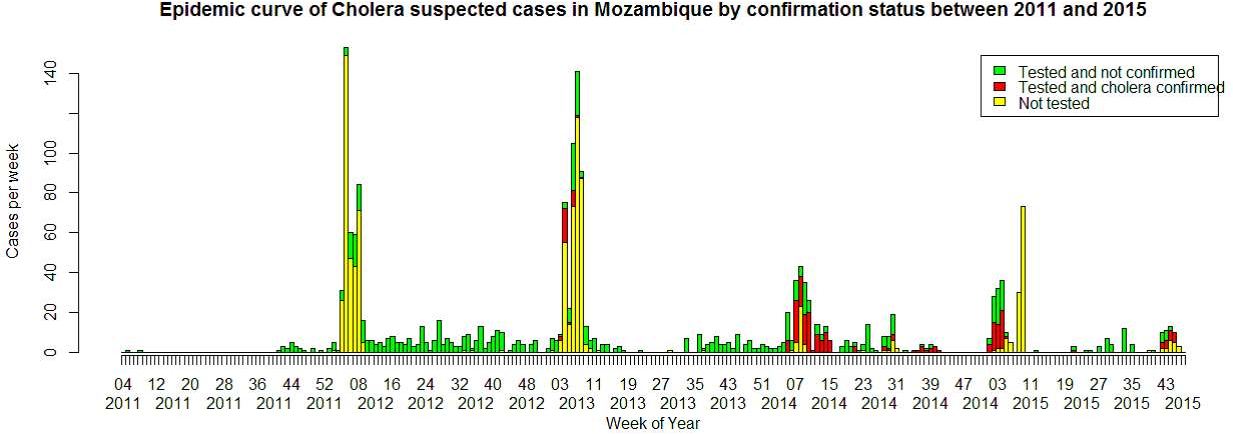
Tested and confirmed cases of cholera through Africhol, Mozambique, 2011–2015.

A total of 972 suspected cases (52.2%) were tested and 228 (23.5%) of them were confirmed (Table 3; Fig 4). The rate of confirmed cholera varied between age groups (p<0.001), with the lowest rate among the age group ≤5 years (26/216, 12%), compared with confirmation rates ranging from 21% to 31% for other age groups. However, after adjusting these results on the surveillance zone, the confirmation rate was no longer associated with age. Only 4.5% of the children 12–23 months who were tested (4/89) were confirmed to be cholera by culture. Among the 207 children aged 2–5 years, 17.3% of those tested (22/127) were culture positive.

**Table 3:** Number of culture tests performed, confirmed cholera cases, and case-fatality ratio by site, 2011–2015, Mozambique.

Characteristics	All zones	Beira	Mocuba	Pemba	Cuamba	Montepuez	Nampula	P value
	N = 1863	N = 427	N = 217	N = 367	N = 233	N = 93	N = 375	
**Culture tests performed**								
No	891 (47.8)	15 (3.5)	11 (5.1)	309 (84.2)	232 (99.6)	92 (98.9)	105 (28.0)	<0.001
Yes	972 (52.2)	412 (96.5)	206 (94.9)	58 (15.8)	1 (0.4)	1 (1.1)	270 (72.0)	
**Cholera confirmed Y/N**	**N = 972**							
No	744 (76.5)	394 (95.6)	197 (95.6)	40 (69.0)	1 (100)	1 (100)	101 (37.4)	<0.001
Yes	228 (23.5)	18 (4.4)	9 (4.4)	18 (31.0)	0	0	169 (62.6)	
**Deaths**	23	2	0	4	6	1	10	
**CFR suspected (%)**	1.2	0.5	0.0	1.1	2.6	1.1	2.7	
**Death among confirmed**	3	0	0	3	-	-	0	
**CFR confirmed (%)**	1.3	0	0	16.7	-	-	0	

CFR: Case fatality ratio.

Cholera confirmed = [culture positive AND oxidase positive] OR [culture positive AND serotype Ogawa].

A total of 23 deaths were identified (CFR suspected, 1.2%), all of which occurred during the rainy season. The CFR was significantly higher in outbreak zones than surveillance sites (2.0% vs 0.6%, p<0.01), and in males compared to females (1.8% vs. 0.6%, p = 0.02). A total of 3 deaths occurred out of 228 cholera confirmed cases (CFR confirmed cases = 1.3%), all of them in Pemba city (Table 3).

### Clinical characteristics of cholera suspected patients

In outbreak zones, most suspected cases were notified from CTCs, whereas in surveillance zones it varied (Table 1). The majority of cases consulted the health facility the same day or the day following symptom onset (70.4%). Most suspected cases were hospitalized (66.1%), except in Beira (5.6%). The majority of cases reported symptoms of watery stools (78.0%), dehydration (66.6%), and vomiting (59.9%), but this varied between sites (S1 Table).

Among all suspected cholera cases, 5% tested HIV positive, but most cases did not have HIV status determined (70%). The highest positivity rate was seen in Beira city (14%). For malaria, 8% of all suspected cholera cases had a positive rapid test result, with a high proportion in Mocuba district (24%), while 62% had unknown malaria status.

### Behaviors and exposures associated with confirmed cholera

Most of the suspected cases had no contact with another suspected case, nor had they attended a funeral or a social event in the seven days prior. The public tap was the most common source of drinking water at home (48.2%), followed by a shallow well (18.3%). The majority of cases reported drinking untreated water (62.3%). Among those who treated water, bleach/chlorine was the most common treatment procedure (38.2%), followed by boiling water (22.3%) (S2 Table).

Factors independently associated with confirmed cholera in the multivariate analysis were: living in Nampula city district, the year 2014, HIV positive status, and the primary water source for drinking (Table 4). Factors associated with hospitalization included: male gender; young age; location; longer duration between date of onset and consultation; presence of rice water stools; vomiting; abdominal pains; leg cramps; HIV positive status; and receiving IV fluids before the consultation (S3 Table). Factors associated with death of suspected cholera cases included: male gender; short duration between disease onset and consultation; rice water stools; abdominal pain; and leg cramps (S4 Table).

**Table 4:** Factors associated with confirmed cholera, 2011–2015, Mozambique—results of the multivariate analysis.

Characteristics	Confirmed cases/tests done (%) n = 948	Adjusted OR	P value
Sex*			
Female	87/418 (20.8)	1	0.33
Male	141/529 (26.7)	1.23 [0.81–1.88]	
Age group*			
1–5	26/210 (12.4)	1	0.47
6–15	48/184 (26.1)	0.90 [0.42–1.91]	
16–25	70/220 (31.8)	0.61 [0.30–1.25]	
26–35	40/140 (28.6)	0.66 [0.30–1.44]	
36–45	21/94 (22.3)	0.47 [0.20–1.12]	
>45	18/82 (22.0)	0.60 [0.25–1.47]	
**Surveillance zones**			
Beira	18/394 (4.6)	1	
Mocuba	9/202 (4.5)	0.69 [0.24–2.00]	<0.001
Nampula	169/270 (62.6)	17.8 [7.8–40.5]	
Pemba	18/58 (31.0)	4.44 [1.75–11.3]	
Other**	14/24 (58.3)	24.5 [5.9–102.1]	
**Year of onset**			
2012	0/293 (0.0)	-	
2013	30/216 (13.9)	1	0.04
2014	130/272 (47.8)	2.12 [1.12–4.00]	
2015	68/167 (40.7)	1.38 [0.62–3.03]	
***HIV status***			
No	42/370 (11.4)	1	0.01
Yes	10/69 (14.5)	4.48 [1.64–12.24]	
Unknown	176/509 (34.6)	1.21 [0.69–2.12]	
**Primary source of drinking water**			
Public tap	125/464 (21.2)	1	<0.01
Shallow well	53/215 (24.7)	0.74 [0.41–1.32]	
Piped water in home	17/52 (32.7)	0.97 [0.43–2.17]	
River/Stream/Lake/Pond	7/44 (15.9)	1.66 [0.49–5.66]	
Other	8/22 (36.4)	4.84 [1.40–16.8]	
Unknown	18/26 (69.2)	5.12 [1.70–15.4]	

Note: a total of eight variables were entered in the complete multivariate model: gender; age group; surveillance zone; year of onset; duration between onset to consultation; HIV status; primary source of drinking water; and treated drinking water.

*Sex and age group were forced in the model.

** Including mainly from Tete, Quelimane and Lichinga districts during outbreaks occurring in 2015.

## Discussion

Our analysis is in line with previous published data showing that cholera in Mozambique is marked by spatial heterogeneity and seasonality, with a high concentration of cases during the rainy period between January and March [5] and inter-epidemic periods largely free of confirmed cases. Each surveillance zone showed a different pattern of suspected cases distributed over time, although there were clear similarities in the seasonality of suspected cases between the surveillance and outbreaks zones. This heterogeneity may be explained by the hypothesis that in Mozambique outbreaks do not evolve locally, but rather follow cholera re-introduction from distant epidemic regions [7]. Our study allowed for the measurement of cholera incidence prospectively. Incidence of suspected cases varied widely between sites and between years within the same site. The incidence based on suspected cases only may be overestimated, given the low culture confirmation rate, as was shown previously in other Africhol countries [9].

There were 23 cholera deaths during the enhanced surveillance period (CFR, 1.2%), similar to what was found previously in Mozambique (0.9% during reported outbreaks in the period 2009–2011). Other studies showed the CFR was likely much higher under non-research conditions when immediate rehydration and transportation to hospitals may not be available [13]. Previous studies in Mozambique using surveillance data showed that 90% of deaths and 70% of cases occurred during the first six weeks of the outbreak [5].

Case distribution by sex and age group showed common patterns in all zones, except for Beira, where a high proportion of suspected cases occurred in persons under five years of age. In these areas, young children with symptoms of acute diarrhea are often brought to healthcare facilities by their mothers for treatment, while adults may be more hesitant to visit healthcare facilities. The proportion of confirmed cases in this age group in Beira was low (3.8%), but was similar to the other age groups from this zone (ranging from 0 to 9%). In another study in Beira, cholera incidence was higher among children below five years of age compared to older age groups [14].

Overall, the proportion of confirmed cases was lower in the one- to five-year-old age group than in older age groups. As we did not have the mandate or resources to study other pathogens that might also be associated with acute diarrhea in young children we cannot ascertain the attributable fraction of cholera to all-cause diarrhea. In the Global Enteric Multicenter Study (GEMS) conducted in four African sites and three Asian sites among children below 5 years, most attributable cases of moderate-to-severe diarrhea were due to four pathogens: *rotavirus*, *Cryptosporidium*, enterotoxigenic *Escherichia coli* producing heat-stable toxin (ST-ETEC), and Shigella [15]. Nonetheless, in one of the study sites located in southern Mozambique (Manhica), with traditionally low cholera incidence, the main pathogens identified among children 2–5 years-old were Shigella (14.9% of all moderate-to-severe diarrhea) and *Vibrio cholerae* (8.3%).

Our results indicate the suspected cases tended to rapidly seek care. This might be explained by the fact that people have the perception that cholera is a serious disease likely to result in death if left untreated. In Beira, there was a very low cholera confirmation and hospitalization. One of the possibilities for this finding is that some of the large outbreaks in Beira might be attributed to other pathogens.

Of the expected known exposures or risk factors, the only one that was independently associated with confirmed cholera in our study was the primary source of drinking water. Drinking water from unknown sources posed a greater risk for cholera. This could indicate wide-spread circulation of *V*.*cholerae* in the drinking water sources of the affected areas during outbreak periods resulting in a higher proportion of common source versus person-to-person transmission. In line with this, most cases occurred during the rainy season (82%), especially in outbreak sites with high incidence, with heavy floods possibly deteriorating water and sanitation system and triggering water borne transmission. Further studies using molecular biology methods, innovative approach to evaluate risk factors for cholera infection (eg. including community controls); small scale spatial epidemiological analysis and other studies such as description of the secondary infection at household and neighbor level should further elucidate the modes of cholera transmission. In parallel, majority of suspected cases reported that they had not attended a mass gathering or market in the seven days before symptom onset. This would underline the over proportional importance of continuous exposure through the water source at the place of residence rather than at isolated mass gathering events. Looking at all factors associated with confirmed cholera, we can see that risk factors are not that different for suspected and confirmed cases. This would reflect the common risk factors for cholera and other water-borne diarrheal diseases.

Although some association between HIV status and confirmed cholera was shown (adjusted OR, 95% CI: 4.5 [1.6–12.2]), this result should be interpreted with caution, because the majority of suspected cases (70%) and confirmed cases (54%) had unknown HIV status. Also, most HIV tests that were carried out and produced positive results came from one site (Beira). Additionally, we could not differentiate any HIV infection from HIV infection with immunosuppression, and HIV infection may simply be a marker for persons with less access to clean water and sanitation. A previous study in Beira indicate that persons with HIV infection had an increased risk of cholera compared to those without HIV infection, although this risk did not reach statistical significance (p = 0.08), and no information on immunosuppression status of enrolled patients was provided in this study [16].

The different cholera characteristics between zones highlight the need to tailor intervention strategies to the specific local setting and at-risk populations and are useful for evidence-based decision making. The data presented here were used to direct interventions to prevent/treat cholera such as timely and solid cholera surveillance system, improved environmental management in particular continued access to safe water and proper sanitation, and the adequate use of cholera vaccines as a complementary immediate measure. The high-risk zones where cholera outbreaks repeatedly occur (e.g., Nampula city in Nampula province, Mocuba district in Zambezia, and Pemba city in Cabo Delgado province) are so-called “cholera hotspots” and may benefit from preventive or reactive cholera immunization campaigns in combination with other cholera control activities (such as WASH activities), as recommended by the World Health Organization (WHO) [17]. The persistence of cholera over decades and the wide-spread risk of drinking contaminated water in those areas will require a decisive comprehensive effort from all stakeholders to improve the situation. No plans for such interventions are yet known and they will likely take years. In contrast OCV can provide rapid protection. A previous OCV campaign had been conducted in Beira city in 2003–2004 and showed a high vaccine effectiveness of one or more doses (78%, 95% CI: 39–92), even in a setting with high HIV prevalence [6]. More recently, in October 2016, another OCV campaign was conducted in Mozambique in Nampula city, targeting high-risk neighborhoods; monitoring and evaluation of this campaign is currently on-going.

Although our study provides valuable information about cholera surveillance in Mozambique, there are some limitations. Only 52.2% of suspected cases were tested and 23.5% were microbiologically confirmed. This limited capacity to confirm cases by culture combined with limited sensitivity of culture lowered the overall sensitivity of our surveillance [9]. Our diagnostic procedures relied exclusively upon culture results, which can be influenced by various factors including collection, transport and storage conditions, training of human resources or previous antibiotic exposure. Also, culture has a limited sensitivity which can translate in a low negative predictive value. It would have been preferable to use PCR testing, however, given resource limitations, we were unable to do so and thus our burden estimations need to be interpreted accordingly. Conversely, acute diarrhea cases might also have been reported as cholera while being caused by other pathogens. There was a high proportion of certain variables missing, which made it difficult to analyze the risk factors associated with confirmed cholera. In addition, data gaps limited our ability to conduct a robust examination of the association of cholera with malaria or HIV, including any differences in associations that may be due to immunosuppression status. The selection criteria for our surveillance included functional surveillance system and functional health structures with access to appropriate case management. Therefore the mortality related estimates cannot be generalized for the entire country which would likely underestimate mortality and CFRs. Moreover, our study did not account for the cholera cases and deaths in the community when the patients did not visit the health facilities. In this analysis we presented cholera cases in Africhol surveillance sites and some cholera outbreaks from 2011–2015. Compared with simultaneous official figures reported by the National Surveillance System, our surveillance system showed fewer cases reported in some of the districts, indicating that the Africhol surveillance was not exhaustive since it was limited to certain surveillance zones. However, the introduction of case-based surveillance methodology into the public-health system has allowed national staff to appreciate its value and expand this approach to other infectious diseases thereby reinforcing national surveillance capacity overall.

## Conclusion

Our study provides the most comprehensive information on cholera in Mozambique available in recent years. This study does not aim to replace the national system but to assess the characteristics of populations at risk of cholera and risk factors in specific sites through an enhanced case-based surveillance. There is a need for continued surveillance, detailed data and stronger laboratory capacity to target prevention and control efforts, including locally adapted WASH interventions and preemptive use of OCV. In order to improve quality and access to safe water and sanitation, the Mozambican government has established investment funds and water supply assets which shall be used for public investment into the water supply system and the management of several companies across Mozambique.

The use of burden data in smaller-scale geographic units will help target higher risk neighborhoods (*bairros*) within the identified districts, and this work is ongoing.

## Supporting information

S1 Table Characteristics of the clinical episode of cholera by study site, 2011–2015, Africhol, Mozambique. (DOCX)

S2 Table Exposures and behaviors of cholera suspected cases by study site, 2011–2015, Africhol, Mozambique. (DOCX)

S3 Table Factors associated with hospitalization of suspected cases, 2011–2015, Africhol, Mozambique—results of the multivariate analysis. (DOCX)

S4 Table Factors associated with death of suspected cases, 2011–2015, Africhol, Mozambique—results of the multivariate analysis. (DOCX)

## References

[1] Bradley M, Shakespeare R, Ruwende A, Woolhouse MEJ, Mason E, Munatsi A. Epidemiological features of epidemic cholera (El Tor) in Zimbabwe. Trans R Soc Trop Med Hyg. 1996;90: 378–382. 8882180 10.1016/s0035-9203(96)90512-x

[2] Mengel MA, Delrieu I, Heyerdahl L, Gessner BD. Cholera outbreaks in Africa. Cholera Outbreaks. 2014; 117–144. 10.1007/82_2014_369 24827501

[3] Gaffga NH, Tauxe RV, Mintz ED. Cholera: A New Homeland in Africa? Am J Trop Med Hyg. 2007;77: 705–713. 17978075

[4] Adagbada AO, Adesida SA, Nwaokorie FO, Niemogha M-T, Coker AO. Cholera Epidemiology in Nigeria: an overview. Pan Afr Med J. 2012;12. PMC3428179 22937199

[5] Gujral L, Sema C, Rebaudet S, Taibo CLA, Manjate AA, Piarroux R, et al Cholera epidemiology in Mozambique using national surveillance data. J Infect Dis. 2013;208 Suppl 1: S107–114. doi: 10.1093/infdis/jit212 24101638 10.1093/infdis/jit212

[6] Lucas ME, Deen JL, von Seidlein L, Wang X-Y, Ampuero J, Puri M, et al Effectiveness of mass oral cholera vaccination in Beira, Mozambique. N Engl J Med. 2005;352: 757–767. doi: 10.1056/NEJMoa043323 15728808 10.1056/NEJMoa043323

[7] Langa JP, Sema C, De Deus N, Colombo MM, Taviani E. Epidemic waves of cholera in the last two decades in Mozambique. J Infect Dev Ctries. 2015;9: 635–641. doi: 10.3855/jidc.6943 26142674 10.3855/jidc.6943

[8] Ansaruzzaman M, Bhuiyan NA, Nair GB, Sack DA, Lucas M, Deen JL, et al Cholera in Mozambique, variant of Vibrio cholerae. Emerg Infect Dis. 2004;10: 2057 doi: 10.3201/eid1011.040682 16010751 10.3201/eid1011.040682 PMC3329043

[9] Sauvageot D, Njanpop-Lafourcade B-M, Akilimali L, Anne J-C, Bidjada P, Bompangue D, et al Cholera Incidence and Mortality in Sub-Saharan African Sites during Multi-country Surveillance. PLoS Negl Trop Dis. 2016;10: e0004679 doi: 10.1371/journal.pntd.0004679 27186885 10.1371/journal.pntd.0004679 PMC4871502

[10] Munier A, Njanpop-Lafourcade B-M, Sauvageot D, Mhlanga RB, Heyerdahl L, Nadri J, et al The African cholera surveillance network (Africhol) consortium meeting, 10–11 June 2015, Lomé, Togo. BMC Proc. 2017;11: 2 doi: 10.1186/s12919-016-0068-z 28813542 10.1186/s12919-016-0068-z PMC5301166

[11] Kaper JB, Morris JG, Levine MM. Cholera. Clin Microbiol Rev. 1995;8: 48–86. 7704895 10.1128/cmr.8.1.48 PMC172849

[12] Smith AM, Njanpop-Lafourcade B-M, Mengel MA, Gessner BD, Sauvageot D, Bidjada B, et al Comparative Characterization of Vibrio cholerae O1 from Five Sub-Saharan African Countries Using Various Phenotypic and Genotypic Techniques. PloS One. 2015;10: e0142989 doi: 10.1371/journal.pone.0142989 26606536 10.1371/journal.pone.0142989 PMC4659613

[13] Sur D, Deen JL, Manna B, Niyogi SK, Deb AK, Kanungo S, et al The burden of cholera in the slums of Kolkata, India: data from a prospective, community based study. Arch Dis Child. 2005;90: 1175–1181. doi: 10.1136/adc.2004.071316 15964861 10.1136/adc.2004.071316 PMC1720149

[14] Deen JL, Von Seidlein L, Sur D, Agtini M, Lucas ME, Lopez AL, et al The high burden of cholera in children: comparison of incidence from endemic areas in Asia and Africa. PLoS Negl Trop Dis. 2008;2: e173 doi: 10.1371/journal.pntd.0000173 18299707 10.1371/journal.pntd.0000173 PMC2254203

[15] Kotloff KL, Nataro JP, Blackwelder WC, Nasrin D, Farag TH, Panchalingam S, et al Burden and aetiology of diarrhoeal disease in infants and young children in developing countries (the Global Enteric Multicenter Study, GEMS): a prospective, case-control study. The Lancet. 2013;382: 209–222. 10.1016/S0140-6736(13)60844-2 23680352

[16] Von Seidlein L, Wang X-Y, Macuamule A, Mondlane C, Puri M, Hendriksen I, et al Is HIV infection associated with an increased risk for cholera? Findings from a case–control study in Mozambique. Trop Med Int Health. 2008;13: 683–688. doi: 10.1111/j.1365-3156.2008.02051.x 18331384 10.1111/j.1365-3156.2008.02051.x

[17] World Health Organization. Cholera vaccines: WHO position paper. Wkly Epidemiol Rec. 2010;85: 117–128. 20349546

