# Supplementary material for: Multi-site cholera surveillance within the African Cholera Surveillance Network shows endemicity in Mozambique, 2011–2015
Source: PLoS Negl Trop Dis. 2017 Oct 9;11(10):e0005941. doi: 10.1371/journal.pntd.0005941 (PMC5648265; doi:10.1371/journal.pntd.0005941)
Supplement: S1 Table — (DOCX) [file pntd.0005941.s001.docx]

**Table S1: Characteristics of the Clinical Episode of Cholera by study site, 2011-2015, Africhol, Mozambique.**

|  |  | **Surveillance Zones** | | | **Outbreak Sites** | | |  |
| --- | --- | --- | --- | --- | --- | --- | --- | --- |
| **Characteristics** | **All zones** | Beira | Mocuba | Pemba | Cuamba | Montepuez | Nampula | P value |
|  | **N=1863** | N=427 | N=217 | N=367 | N=233 | N=93 | N=375 |  |
| ***Delay between symptom onset and consultation*** |  |  |  |  |  |  |  |  |
| 0 day | 743 (39.9) | 79 (18.5) | 62 (28.6) | 215 (58.6) | 114 (48.9) | 36 (38.7) | 165 (44.0) | <0.001 |
| 1 day | 569 (30.5) | 179 (41.9) | 73 (33.6) | 91 (24.8) | 58 (24.9) | 6 (6.5) | 111 (29.6) |  |
| 2-4 days | 388 (20.8) | 131 (30.7) | 59 (27.2) | 37 (10.1) | 32 (13.7) | 18 (19.4) | 87 (23.2) |  |
| >= 5 days | 130 (7.0) | 31 (7.3) | 20 (9.2) | 10 (2.7) | 27 (11.6) | 31 (33.3) | 7 (1.9) |  |
| *Missing* | 33 (1.8) | 7 (1.6) | 3 (1.4) | 14 (3.8) | 2 (0.9) | 2 (2.1) | 5 (1.3) |  |
| ***Symptoms*** |  |  |  |  |  |  |  |  |
| Acute Diarrhea | 1806 (96.9) | 421 (98.6) | 216 (99.5) | 363 (98.9) | 228 (97.9) | 65 (69.9) | 364 (97.1) | 0.71 |
| Watery Stools | 1454 (78.0) | 367 (86.0) | 201 (92.6) | 330 (89.9) | 117 (50.2) | 55 (59.1) | 240 (64.0) | <0.001 |
| Rice water stools | 320 (17.2) | 36 (8.4) | 21 (9.7) | 11 (3.0) | 4 (1.7) | 5 (5.4) | 191 (50.9) | <0.001 |
| Vomiting | 1117 (59.9) | 221 (51.8) | 116 (53.5) | 313 (85.3) | 107 (45.9) | 42 (45.2) | 186 (49.6) | <0.001 |
| Dehydration | 1241 (66.6) | 205 (48.0) | 124 (57.1) | 302 (82.3) | 97 (41.6) | 82 (88.2) | 300 (80.0) | <0.001 |
| Abdominal pain | 427 (22.9) | 106 (24.8) | 83 (38.3) | 24 (6.5) | 1 (0.4) | 8 (8.6) | 173 (46.1) | <0.001 |
| Leg cramps | 155 (8.3) | 70 (16.4) | 5 (2.3) | 15 (4.0) | 0 | 1 (1.1) | 62 (16.5) |  |
| ***HIV status*** |  |  |  |  |  |  |  | 0.001 |
| Negative | 466 (25.0) | 250 (58.6) | 60 (27.7) | 77 (21.0) | 1 (0.4) | 4 (4.3) | 68 (18.1) |  |
| Positive | 96 (5.2) | 59 (13.8) | 15 (6.9) | 10 (2.7) | 2 (0.9) | 3 (3.2) | 3 (0.8) |  |
| Unknown | 1301 (69.8) | 118 (27.6) | 142 (65.4) | 280 (76.3) | 230 (98.7) | 86 (92.5) | 304 (81.1) |  |
| ***Malaria status*** |  |  |  |  |  |  |  | <0.001 |
| Negative | 553 (29.7) | 205 (48.0) | 79 (36.4) | 76 (20.7) | 8 (3.4) | 6 (6.4) | 160 (42.7) |  |
| Positive | 149 (8.0) | 44 (10.3) | 52 (24.0) | 19 (5.2) | 3 (1.3) | 1 (1.1) | 11 (2.9) |  |
| Unknown | 1161 (62.3) | 178 (41.7) | 86 (39.6) | 272 (74.1) | 222 (95.3) | 86 (92.5) | 204 (54.4) |  |
| ***Hospitalization*** |  |  |  |  |  |  |  | <0.001 |
| No | 620 (33.3) | 400 (93.7) | 94 (43.3) | 11 (3.0) | 11 (4.7) | 13 (14.0) | 74 (19.7) |  |
| Yes | 1232 (66.1) | 24 (5.6) | 123 (56.7) | 353 (96.2) | 222 (95.3) | 79 (84.9) | 300 (80.0) |  |
| Unknown | 11 (0.6) | 3 (0.7) | 0 | 3 (0.8) | 0 | 1 (1.1) | 1 (0.3) |  |
| ***Patient outcome*** |  |  |  |  |  |  |  | 0.015 |
| Alive | 1839 (98.7) | 425 (99.5) | 217 (100) | 363 (98.9) | 227 (97.4) | 92 (98.9) | 364 (97.1) |  |
| Dead | 23 (1.2) | 2 (0.5) | 0 | 4 (1.1) | 6 (2.6) | 1 (1.1) | 10 (2.7) |  |
| Unknown | 1 (0.05) | 0 | 0 | 0 | 0 | 0 | 1 (0.2) |  |
