# Supplementary material for: Multi-site cholera surveillance within the African Cholera Surveillance Network shows endemicity in Mozambique, 2011–2015
Source: PLoS Negl Trop Dis. 2017 Oct 9;11(10):e0005941. doi: 10.1371/journal.pntd.0005941 (PMC5648265; doi:10.1371/journal.pntd.0005941)
Supplement: S2 Table — (DOCX) [file pntd.0005941.s002.docx]

**Table S2: Exposures and Behaviors of Cholera Suspected Cases by study site, 2011-2015, Africhol, Mozambique**

|  |  | **Surveillance Zones** | | | **Outbreak Sites** | | |  |
| --- | --- | --- | --- | --- | --- | --- | --- | --- |
| **Risk Factors** | **All zones (%total cases)** | Beira (%tot) | Mocuba (%tot) | Pemba (%tot) | Cuamba (%tot) | Montepuez (%tot) | Nampula (%tot) | P value (between sites) |
|  | **N=1863** | N=427 | N=217 | N=367 | N=233 | N=93 | N=375 |  |
| Had contact with another known or reported suspected cholera case |  |  |  |  |  |  |  |  |
| No | 1280 (68.7) | 399 (93.4) | 207 (95.4) | 247 (67.3) | 9 (3.8) | 4 (4.3) | 281 (74.9) | <0.001 |
| Yes | 38 (2.0) | 0 | 0 | 7 (1.9) | 2 (0.9) | 1 (1.1) | 25 (6.7) |  |
| Unknown | 545 (29.3) | 28 (6.6) | 10 (4.6) | 113 (30.8) | 222 (95.3) | 88 (94.6) | 69 (18.4) |  |
| Attended or participated in a funeral in the last seven days |  |  |  |  |  |  |  |  |
| No | 1289 (69.2) | 388 (90.9) | 198 (91.2) | 256 (69.8) | 11 (4.7) | 7 (7.5) | 294 (78.4) | <0.001 |
| Yes | 23 (1.2) | 3 (0.7) | 9 (4.2) | 0 | 2 (0.9) | 0 | 9 (2.4) |  |
| Unknown | 551 (29.6) | 36 (8.4) | 10 (4.6) | 111 (30.2) | 220 (94.4) | 86 (92.5) | 72 (19.2) |  |
| Attended a social gathering or event in the last seven days |  |  |  |  |  |  |  |  |
| No | 1263 (67.8) | 369 (86.4) | 192 (88.5) | 254 (69.2) | 12 (5.2) | 4 (4.3) | 295 (78.7) | <0.001 |
| Yes | 48 (2.6) | 25 (5.9) | 15 (6.9) | 3 (0.8) | 1 (0.4) | 1 (1.1) | 3 (0.8) |  |
| Unknown | 552 (29.7) | 31 (7.7) | 10 (4.6) | 110 (30.0) | 220 (94.4) | 88 (94.6) | 77 (20.5) |  |
| Attended a market or trading center in the last seven days |  |  |  |  |  |  |  |  |
| No | 1084 (58.2) | 244 (57.1) | 173 (79.7) | 246 (67.0) | 11 (4.7) | 5 (5.4) | 271 (72.3) | <0.001 |
| Yes | 225 (12.1) | 151 (35.4) | 34 (15.7) | 8 (2.2) | 0 | 2 (2.1) | 29 (7.7) |  |
| Unknown | 554 (29.7) | 32 (7.5) | 10 (4.6) | 113 (30.8) | 222 (95.3) | 86 (92.5) | 75 (20.0) |  |
| Traveled outside the home village/town in the last seven days |  |  |  |  |  |  |  |  |
| No | 1229 (66.0) | 355 (83.1) | 186 (85.7) | 247 (67.3) | 12 (5.2) | 5 (5.4) | 290 (77.4) | 0.03 |
| Yes | 39 (2.1) | 19 (4.5) | 10 (4.6) | 3 (0.8) | 1 (0.4) | 0 | 5 (1.3) |  |
| Unknown | 595 (31.9) | 53 (12.4) | 21 (9.7) | 117 (31.9) | 220 (94.4) | 88 (94.6) | 80 (21.3) |  |
| Primary source of drinking water at home |  |  |  |  |  |  |  |  |
| Public Tap | 898 (48.2) | 380 (89.0) | 60 (27.6) | 121 (33.0) | 8 (3.4) | 36 (38.7) | 217 (57.9) | <0.001 |
| Shallow well | 342 (18.3) | 12 (2.8) | 113 (52.1) | 54 (14.7) | 5 (2.2) | 20 (21.5) | 96 (25.6) |  |
| Piped water in home/yard | 136 (7.3) | 24 (5.6) | 1 (0.5) | 77 (21.0) | 0 | 0 | 34 (9.1) |  |
| River/Stream/Lake/Pond | 88 (4.7) | 1 (0.2) | 32 (14.8) | 13 (3.5) | 1 (0.4) | 12 (12.9) | 3 (0.8) |  |
| Other | 277 (14.9) | 6 (1.4) | 9 (4.1) | 19 (5.2) | 219 (94.0) | 14 (15.1) | 9 (2.4) |  |
| *missing* | 122 (6.6) | 4 (1.0) | 2 (0.9) | 83 (22.6) | 0 | 11 (11.8) | 16 (4.3) |  |
| Drinking water treated |  |  |  |  |  |  |  |  |
| No | 1160 (62.3) | 293 (68.6) | 148 (68.2) | 224 (61.0) | 11 (4.7) | 40 (43.0) | 304 (81.1) | <0.001 |
| Yes | 220 (11.8) | 113 (26.5) | 44 (20.3) | 33 (9.0) | 3 (1.3) | 3 (3.2) | 24 (6.4) |  |
| Unknown | 483 (25.9) | 21 (4.9) | 25 (11.5) | 110 (30.0) | 219 (94.0) | 50 (53.8) | 47 (12.5) |  |
| Drinking water treatment* |  |  |  |  |  |  |  |  |
| Bleach/Chlorine | 84 (38.2) | 52 (46.0) | 22 (50.0) | 3 (9.1) | 2 (66.7) | 1 (33.3) | 4 (16.7) | <0.001 |
| Boiled | 49 (22.3) | 33 (29.2) | 5 (11.4) | 5 (15.2) | 1 (33.3) | 0 | 5 (20.8) |  |
| Filter | 10 (4.5) | 7 (6.2) | 3 (6.8) | 0 | 0 | 0 | 0 |  |
| Other | 13 (5.9) | 9 (8.0) | 3 (6.8) | 1 (3.0) | 0 | 0 | 0 |  |
| Unknown | 64 (29.1) | 12 (10.6) | 11 (25.0) | 24 (72.7) | 0 | 2 (66.7) | 15 (62.5) |  |

*Among n=220 cases using treated water.
