# Supplementary material for: Multi-site cholera surveillance within the African Cholera Surveillance Network shows endemicity in Mozambique, 2011–2015
Source: PLoS Negl Trop Dis. 2017 Oct 9;11(10):e0005941. doi: 10.1371/journal.pntd.0005941 (PMC5648265; doi:10.1371/journal.pntd.0005941)
Supplement: S3 Table — (DOCX) [file pntd.0005941.s003.docx]

**Table S3: Factors Associated with Hospitalization of Suspected Cases, 2011-2015, Africhol, Mozambique - Results of the Multivariate Analysis**

| **Characteristics** | **Hospitalizations**  **(inpatients)**  **No. (%)** | **Adjusted OR [95%CI]** | **P value** |
| --- | --- | --- | --- |
| ***Gender*** |  |  |  |
| Female | 581/870 (66.8) | 1 | 0.003 |
| Male | 650/988 (65.8) | 1.82 [1.22-2.70] |  |
| ***Age group*** |  |  |  |
| 0-5 | 128/308 (41.6) | 2.18 [1.01-4.72] | 0.02 |
| 6-15 | 280/387 (72.4) | 2.55 [1.26-5.15] |  |
| 16-25 | 362/478 (75.7) | 1.41 [0.73-2.73] |  |
| 26-35 | 189/286 (66.1) | 1.09 [0.54-2.23] |  |
| 36-45 | 115/181 (63.5) | 0.96 [0.45-2.03] |  |
| >45 | 131/188 (69.7) | 1 |  |
| ***Surveillance zone*** |  |  |  |
| Beira | 24/427 (5.6) | 0.002 [0.0009-0.006] | <0.001 |
| Cuamba | 222/233 (95.3) | 116 [31-432] |  |
| Mocuba | 123/217 (56.7) | 0.09 [0.05-0.19] |  |
| Montepuez | 79/93 (85.0) | 9.61 [3.10-29.77] |  |
| Nampula | 300/375 (80.0) | 1 |  |
| Pemba | 353/367 (96.2) | 19.5 [7.2-52.7] |  |
| Other | 131/151 (86.8) | 0.04 [0.01-0.14] |  |
| ***Year*** |  |  |  |
| 2011 | 4/22 (18.2) | 1.42 [0.32-6.34] | <0.001 |
| 2012 | 377/640 (58.9) | 1 |  |
| 2013 | 407/579 (70.3) | 0.51 [0.26-0.99] |  |
| 2014 | 161/316 (51.0) | 0.63 [0.30-1.31] |  |
| 2015 | 283/306 (92.5) | 52.3 [14.7-185.8] |  |
| ***Duration onset to consultation*** |  |  |  |
| 0 day | 547/746 (73.6) | 1 | 0.04 |
| 1 day | 343/569 (60.3) | 1.78 [1.08-2.95] |  |
| 2-4 days | 235/388 (60.6) | 2.28 [1.24-4.18] |  |
| >=5 days | 85/130 (65.4) | 1.44 [0.65-3.19] |  |
| ***Rice water stools*** |  |  |  |
| No | 698/1228 (56.8) | 1 | <0.001 |
| Yes | 276/320 (86.3) | 2.65 [1.34-5.28] |  |
| Unknown | 258/313 (82.4) | 0.35 [0.15-0.85] |  |
| ***Vomiting*** |  |  |  |
| No | 166/432 (38.4) | 1 | <0.001 |
| Yes | 843/1117 (75.5) | 1.84 [1.11-3.07] |  |
| Unknown | 223/314 (71.0) | 0.64 [0.33-1.24] |  |
| ***Abdominal pain*** |  |  |  |
| No | 329/673 (48.9) | 1 | <0.001 |
| Yes | 297/427 (69.6) | 2.53 [1.48-4.33] |  |
| Unknown | 606/763 (79.4) | 0.83 [0.31-2.21] |  |
| ***Leg cramps*** |  |  |  |
| No | 510/916 (55.7) | 1 | <0.001 |
| Yes | 92/155 (59.4) | 3.60 [1.52-8.53] |  |
| Unknown | 630/792 (79.6) | 0.42 [0.15-1.15] |  |
| ***HIV Status*** |  |  |  |
| Negative | 201/466 (43.1) | 1 | <0.001 |
| Positive | 41/96 (42.7) | 2.64 [1.09-6.42] |  |
| Unknown | 990/1301 (66.1) | 0.43 [0.24-0.78] |  |
| ***IV fluids received before the consultation*** |  |  |  |
| No | 74/113 (65.5) | 1 | <0.001 |
| Yes | 252/263 (95.8) | 7.31 [1.77-30.23] |  |
| Unknown | 906/1487 (60.9) | 0.67 [0.26-1.77] |  |
| ***Attended a market or trading center in the last seven days*** |  |  |  |
| No | 729/1084 (67.3) | 1 | 0.01 |
| Yes | 62/225 (27.6) | 0.46 [0.23-0.89] |  |
| Unknown | 441/554 (79.6) | 0.49 [0.27-0.89] |  |

Note: A total of 27 variables were entered in the complete multivariate model: gender; age group; surveillance zone; year of onset; duration onset to consultation; diarrhea count category; watery stools; rice water stools; nausea; vomiting; dry mucous membranes; dehydration; abdominal pain; leg cramps; HIV status; oral rehydration salts (ORS); intravenous (IV) fluids and antibiotics received before the consultation; ORS and IV fluids received during the consultation; attended/participated in a funeral, a social gathering, a market in the last seven days; traveled outside town in the last seven days; primary source of drinking water; treated drinking water; culture test done.
