# Supplementary material for: Multi-site cholera surveillance within the African Cholera Surveillance Network shows endemicity in Mozambique, 2011–2015
Source: PLoS Negl Trop Dis. 2017 Oct 9;11(10):e0005941. doi: 10.1371/journal.pntd.0005941 (PMC5648265; doi:10.1371/journal.pntd.0005941)
Supplement: S4 Table — (DOCX) [file pntd.0005941.s004.docx]

**Table S4: Factors Associated with Death of Suspected Cases, 2011-2015, Africhol, Mozambique - Results of the Multivariate Analysis**

| **Characteristics** | **Deaths**  **No. (%)** | **Adjusted OR [95%CI]** | **P value** |
| --- | --- | --- | --- |
| ***Gender**** |  |  |  |
| Female | 5/870 (0.6) | 1 | 0.04 |
| Male | 18/988 (1.8) | 3.10 [1.06-9.05] |  |
| ***Age group**** |  |  |  |
| 0-5 | 3/308 (0.97) | 1 | 0.43 |
| 6-15 | 1/387 (0.26) | 0.12 [0.01-1.28] |  |
| 16-25 | 6/478 (1.26) | 0.32 [0.07-1.48] |  |
| 26-35 | 5/286 (1.75) | 0.46 [0.08-2.48] |  |
| 36-45 | 4/181 (2.21) | 0.51 [0.08-3.10] |  |
| >45 | 4/188 (2.1) | 0.78 [1.15-4.15] |  |
| ***Duration: onset to consultation*** |  |  |  |
| 0 day | 16/743 (2.15) | 1 | <0.01 |
| 1 day | 1/569 (0.18) | 0.06 [0.007-0.50] |  |
| 2-4 days | 3/388 (0.77) | 0.24 [0.06-0.91] |  |
| >=5 days | 1/130 (0.77) | 0.15 [0.02-1.43] |  |
| ***Rice water stools*** |  |  |  |
| No | 4/1228 (0.33) | 1 | 0.01 |
| Yes | 10/320 (3.13) | 4.78 [1.31-17.43] |  |
| Unknown | 9/313 (2.88) | 6.30 [1.67-23.80] |  |
| ***Abdominal pain*** |  |  |  |
| Unknown | 11/763 (1.44) | 1 | <0.001 |
| No | 1/673 (0.15) | 3.16 [0.30-33.35] |  |
| Yes | 11/427 (2.58) | 12.3 [3.60-42.06] |  |
| ***Leg cramps*** |  |  |  |
| No | 1/916 (0.11) | 1 | <0.01 |
| Yes | 4/155 (2.58) | 15.1 [1.5-156.7] |  |
| Unknown | 18/792 (2.27) | 33.7 [3.6-314.9] |  |

Note: A total of 10 variables were entered in the full multivariate model: gender; age group; surveillance zone; duration onset to consultation; rice water stools; abdominal pain; leg cramps; hospitalization; attended a market in the last seven days; and primary source of drinking water. *Sex and age group were forced in the model.
